# A randomized phase 3 study of ixazomib–dexamethasone versus physician’s choice in relapsed or refractory AL amyloidosis

**DOI:** 10.1038/s41375-021-01317-y

**Published:** 2021-06-24

**Authors:** Angela Dispenzieri, Efstathios Kastritis, Ashutosh D. Wechalekar, Stefan O. Schönland, Kihyun Kim, Vaishali Sanchorawala, Heather J. Landau, Fiona Kwok, Kenshi Suzuki, Raymond L. Comenzo, Deborah Berg, Guohui Liu, Arun Kumar, Douglas V. Faller, Giampaolo Merlini

**Affiliations:** 1 Division of Hematology, Mayo Clinic, Rochester, NY USA; 2 Department of Clinical Therapeutics, National and Kapodistrian University of Athens, School of Medicine, Athens, Greece; 3 National Amyloidosis Centre, Royal Free London NHS Foundation Trust, University College London, London, UK; 4 Department of Medicine V (Hematology, Oncology and Rheumatology), Amyloidosis Center Heidelberg, Heidelberg University Hospital, Heidelberg, Germany; 5 Division of Hematology/Oncology, Department of Medicine, Samsung Medical Center, Sungkyunkwan University School of Medicine, Seoul, Korea; 6 Amyloidosis Center, Boston University School of Medicine, Boston Medical Center, Boston, MA USA; 7 Memorial Sloan Kettering Cancer Center, New York, NY USA; 8 Clinical Haematology, Westmead Hospital, Sydney, NSW Australia; 9 Department of Hematology, Japanese Red Cross Medical Center, Tokyo, Japan; 10 John C. Davis Myeloma and Amyloid Program, Tufts Medical Center, Boston, MA USA; 11 Millennium Pharmaceuticals, Inc., a wholly owned subsidiary of Takeda Pharmaceutical Company Limited, Cambridge, MA USA; 12 Amyloidosis Research and Treatment Center, Foundation IRCCS Policlinico San Matteo, Department of Molecular Medicine, University of Pavia, Pavia, Italy

**Keywords:** Phase III trials, Cancer therapy, Myeloma

## Abstract

In the first phase 3 study in relapsed/refractory AL amyloidosis (TOURMALINE-AL1 NCT01659658), 168 patients with relapsed/refractory AL amyloidosis after 1–2 prior lines were randomized to ixazomib (4 mg, days 1, 8, 15) plus dexamethasone (20 mg, days 1, 8, 15, 22; *n* = 85) or physician’s choice (dexamethasone ± melphalan, cyclophosphamide, thalidomide, or lenalidomide; *n* = 83) in 28-day cycles until progression or toxicity. Primary endpoints were hematologic response rate and 2-year vital organ deterioration or mortality rate. Only the first primary endpoint was formally tested at this interim analysis. Best hematologic response rate was 53% with ixazomib–dexamethasone vs 51% with physician’s choice (*p* = 0.76). Complete response rate was 26 vs 18% (*p* = 0.22). Median time to vital organ deterioration or mortality was 34.8 vs 26.1 months (hazard ratio 0.53; 95% CI, 0.32–0.87; *p* = 0.01). Median treatment duration was 11.7 vs 5.0 months. Adverse events of clinical importance included diarrhea (34 vs 30%), rash (33 vs 20%), cardiac arrhythmias (26 vs 15%), nausea (24 vs 14%). Despite not meeting the first primary endpoint, all time-to-event data favored ixazomib–dexamethasone. These results are clinically relevant to this relapsed/refractory patient population with no approved treatment options.

## Introduction

Immunoglobulin light chain systemic light-chain (AL) amyloidosis is a rare clonal plasma-cell disorder that produces misfolded immunoglobulin light-chain proteins, which aggregate and deposit as amyloid fibrils in tissues/organs, leading to multi-organ dysfunction [1–3]. The most frequently involved organs are the heart and kidney [1, 4]. Prognosis is generally poor: in patients with advanced cardiac involvement, survival is limited to 4–6 months [4, 5]. Among all patients, 24% will die within 6 months of diagnosis and only 5–31% will survive for 10 years [6–10]. Autologous stem cell transplant achieves the best long-term outcomes for AL amyloidosis patients, although most patients are ineligible due to poor physical condition [11–15].

When this trial was designed, there were no approved or standard-of-care treatments for AL amyloidosis. Since completion of this trial, daratumumab has been approved as part of a cyclophosphamide, bortezomib, dexamethasone treatment for newly diagnosed patients [16]. No drug is approved for AL amyloidosis as second line therapy. Management of AL amyloidosis is primarily based on off-label use of therapies for multiple myeloma (MM) [11, 17], and with these treatments, deeper hematologic responses have been associated with organ responses and prolonged overall survival (OS) [3, 11, 18, 19]. Proteasome inhibitor (PI)-based combinations have demonstrated activity in newly diagnosed and relapsed/refractory AL amyloidosis [12, 15, 20]; however, treatment with certain PIs may be challenging due to burden of administration, comorbidities, organ dysfunction, or toxicity concerns [12, 15]. New, active treatments that are tolerable in the context of multi-organ dysfunction are needed.

TOURMALINE-AL1 is the first phase 3 study in relapsed/refractory AL amyloidosis and investigates ixazomib–dexamethasone vs physician’s choice of treatment. The oral PI ixazomib is approved in more than 65 countries in combination with lenalidomide–dexamethasone to treat MM after ≥1 prior therapy [21–23]. In a phase 1/2 study in relapsed/refractory AL amyloidosis, ixazomib appeared active and well tolerated in 27 patients (10 of whom received added dexamethasone) with a hematologic response rate of 52% (100% in PI-naïve patients [*n* = 5]) and organ response rate of 56% [24]. Here we report results of TOURMALINE-AL1 in order to further our understanding of treatment of this rare disease.

## Patients and methods

### Study design

TOURMALINE-AL1 is a phase 3, randomized, open-label study conducted in 68 sites across 19 countries in Europe, North America, Latin America, and Asia-Pacific. Adult patients with biopsy-proven AL amyloidosis and major organ (cardiac and/or renal) involvement by International Society of Amyloidosis (ISA) criteria [25] were eligible. Patients had to have relapsed/refractory disease after 1–2 prior therapies. Patients could have prior exposure but not be refractory to PI therapy. Full eligibility criteria are in the Supplementary Appendix.

The study was conducted in accordance with Good Clinical Practice and applicable regulatory requirements. Local ethics committees/institutional review boards approved the protocol. Patients provided written informed consent. Data were analyzed by the sponsor; authors had access to the data. During the study, the sponsor and the trial-monitoring component of the contract research organization, and the Independent Review Committee remained blinded to the efficacy data. The trial was registered at ClinicalTrials.gov as NCT01659658.

### Procedures

Patients were randomized 1:1 to receive oral ixazomib (4 mg) on days 1, 8, and 15 plus oral dexamethasone 20 mg/day on days 1, 8, 15, and 22, or physician’s choice in 28-day cycles.

For physician’s choice, investigators selected a dexamethasone-based non-PI-containing regimen from a prespecified list; the choice for each screened patient was made prior to randomization. The choices included dexamethasone alone 20 mg/day by mouth (PO), days 1–4, 9–12, and 17–20; dexamethasone 20 mg/day PO, days 1–4, plus melphalan 0.22 mg/kg PO, days 1–4; dexamethasone 20 mg/day PO, days 1, 8, 15, and 22, plus cyclophosphamide 500 mg PO, days 1, 8, and 15; dexamethasone 20 mg/day PO, days 1, 8, 15, and 22, plus thalidomide 50–200 mg/day PO (starting dose 50 mg/day, increased as tolerated); dexamethasone 20 mg/day PO, days 1, 8, 15, and 22, plus lenalidomide 15 mg/day, days 1–21. Regimens were given in 28-day cycles with dose adjustments based on toxicities; dexamethasone could be increased up to 40 mg/day on days 1, 8, 15, and 22 after 4 weeks, if the lower dose was tolerated without grade >2 dexamethasone-related toxicities.

Patients were treated until disease progression/death or unacceptable toxicity, or study termination, whichever occurred first. Patients receiving melphalan–dexamethasone were treated until best response (defined as a the deepest response achieved) plus 2 additional cycles, or to a maximum of 18 months of therapy or 600 mg total dose of melphalan, given the increased risk of second primary malignancies with prolonged melphalan use noted in the melphalan package insert [26]. Crossover from physician’s choice to ixazomib–dexamethasone was not permitted.

Conduct was overseen by a steering committee. Randomization was stratified by Mayo 2004 cardiac risk stage (1 vs 2 vs a subgroup of 3, based on cardiac biomarkers) [5], relapsed vs refractory disease, and prior PI exposure (naïve vs exposed). The randomization scheme was generated by an independent statistician, with treatment assignment via an Interactive Voice Response System.

### Outcomes and assessments

The original co-primary endpoints were best overall hematologic response rate (partial response or better), based on central laboratory results and 2010 ISA criteria [19], and OS. After discussion with regulatory agencies, and considering the methods by which patients are treated and the intention to demonstrate a meaningful measure of clinical benefit, OS was replaced by the novel endpoint of 2-year vital organ (heart and kidney) deterioration or mortality rate as the co-primary endpoint. Cardiac deterioration was defined as the need for hospitalization for heart failure; kidney deterioration was defined as progression to end-stage renal disease requiring maintenance dialysis or renal transplantation. Key secondary endpoints were OS, and hematologic complete response (CR) rate by central laboratory results and ISA criteria. Other secondary endpoints included: hematologic progression-free survival (PFS), vital organ (heart/kidney) PFS, overall (hematologic and/or vital organ) PFS, time to vital organ deterioration and mortality, vital organ best response, duration of hematologic response, time to treatment failure (death, hematologic or major organ progression, hematologic response with stable but clinically morbid organ disease requiring additional therapy, or withdrawal for any reason) time to subsequent anticancer treatment, adverse events (AEs), and serious AEs (SAEs). Full details are provided in the Supplementary Appendix.

Response was evaluated according to central laboratory results and the ISA’s Revised Consensus Response Criteria, as evaluated by a blinded adjudication committee [19]. Hematologic response was assessed every cycle from the date of first dose until end of treatment and every 6 weeks thereafter until documented progression or initiation of subsequent therapy. Organ response was assessed after cycles 3, 6, 9, and 12 and then every 3 cycles thereafter until disease progression or the initiation of subsequent therapy. Best response was defined as a plateau in deepest response achieved. After disease progression, patients were followed for overall survival and subsequent therapy at least every 12 weeks. AEs were graded according to the National Cancer Institute’s Common Terminology Criteria for Adverse Events, version 4.03. AEs were assessed from first dose through 30 days after administration of the last dose of study drug or the start of subsequent anticancer therapy, whichever occurred first. After consultation with regulatory authorities, the protocol was amended in July 2015 to exclude enrollment of PI-exposed patients, to balance the treatment arms, with a target ratio of approximately 50:50 PI-exposed vs PI-naïve patients.

### Statistical analysis

The 2 primary endpoints and key secondary endpoints were planned to be tested sequentially to control family-wise error rate. Assuming a hazard ratio (HR) of 0.63, a total sample size of 248 (145 death events) was determined to give 80% power to test OS at a 2-sided alpha of 0.05. As this sample size also gave adequate power to test the endpoints of overall hematologic response rate and 2-year vital organ deterioration or mortality rate, which later became the co-primary endpoints, sample size based on the OS endpoint was retained as the final sample size for the study. There were two planned interim analyses (IA) and one final analysis. The first IA (IA1) was planned when ~176 patients (power was 90%, assuming 65 and 40% responses in ixazomib–dexamethasone and physicians’ choice arms; see Supplementary methods for justification of assumptions) had completed 6 cycles or had discontinued beforehand to test hematologic response at 0.04 alpha and 2-year vital organ deterioration and mortality rate at 0.01. The second IA was planned when 218 patients had completed 2 years of treatment or discontinued, to test 2-year vital organ deterioration and mortality rate with appropriate alpha carried over from IA1. The final IA was to test OS followed by hematologic CR rate with appropriate alpha carried over from the second IA. As the study did not meet the first primary endpoint at IA1, conducted with data from 168 patients, enrollment was stopped on June 5, 2019.

All efficacy data are reported for the intent-to-treat population. For binary endpoints, arms were compared using an unstratified Cochran–Mantel–Haenszel (CMH) test; a logistic regression model was used to estimate treatment effect and two-sided 95% confidence intervals (CIs). For time-to-event endpoints, arms were compared using a two-sided, stratified log-rank test; an unadjusted stratified Cox model was used to estimate HRs and two-sided 95% CIs. Safety data are reported descriptively in the safety population (patients who received ≥1 dose). Missing data were treated as missing with no data imputation; for the primary endpoints, missing data were counted as failures. SAS version 9.1 (or higher) was used for all analyses.

## Results

### Patients

Between December 2012 and August 2018, 168 patients were randomized to receive ixazomib–dexamethasone (*n* = 85) or physician’s choice (*n* = 83) (Supplementary Fig. 1). Physician’s choice regimens were lenalidomide–dexamethasone (*n* = 47), melphalan–dexamethasone (*n* = 24), cyclophosphamide–dexamethasone (*n* = 10), and thalidomide–dexamethasone (*n* = 2); no patients received dexamethasone alone. Baseline characteristics were well balanced between treatment arms (Table 1).

**Table 1:** Patient demographics and baseline disease characteristics (intent-to-treat population).

	Ixazomib–dexamethasone (*n* = 85)	Physician’s choice (*n* = 83)
Median age, years (range)	65 (38–84)	66 (33–82)
Age category		
<65 years	39 (46)	38 (46)
65–75 years	37 (44)	35 (42)
≥75 years	9 (11)	10 (12)
Male	51 (60)	46 (55)
Race^a^		
White	70 (82)	70 (84)
Black or African American	1 (1)	0
Asian	11 (13)	13 (16)
Region		
North America	29 (34)	22 (27)
Europe	37 (44)	39 (47)
Rest of the world	19 (22)	22 (27)
ECOG performance status		
0	36 (42)	34 (41)
1	41 (48)	38 (46)
2	8 (9)	11 (13)
Sites of amyloid involvement^b^		
Heart	53 (62)	59 (71)
Liver	9 (11)	8 (10)
Kidney	60 (71)	48 (58)
Gastrointestinal tract	11 (13)	12 (14)
Lung	1 (1)	3 (4)
Autonomic nerve	5 (6)	5 (6)
Peripheral nerve	10 (12)	8 (10)
Skin	4 (5)	3 (4)
Muscle tissue	0	1 (1)
Tongue	6 (7)	6 (7)
Carpal tunnel syndrome	3 (4)	1 (1)
Other sites	13 (15)	13 (16)
Heart/kidney involvement		
Both	28 (33)	24 (29)
Heart (no kidney)	25 (29)	35 (42)
Kidney (no heart)	32 (38)	24 (29)
Median sites of amyloid involvement at diagnosis, *n* (range)	2 (1–5)	2 (1–7)
Mayo cardiac risk stage^c^		
I	27 (32)	26 (31)
II	41 (48)	43 (52)
III	17 (20)	14 (17)
NYHA Class		
0 and I	54 (64)	52 (63)
II and III	31 (36)	31 (37)
Serum creatinine clearance		
<60 mL/min	37 (44)	30 (36)
≥60 mL/min	48 (56)	53 (64)
Median time from diagnosis, months (range)	34.5 (4.2–196.1)	32.6 (2.1–114.5)
Relapsed/refractory to last prior therapy	68 (80)/17 (20)	66 (80)/17 (20)
Prior lines of therapy or SCT		
≤1	50 (59)	50 (60)
≥2	35 (41)	33 (40)
PI-naïve/exposed	46 (54)/39 (46)	44 (53)/39 (47)
Received prior transplant	40 (47)	31 (37)
Type of prior therapy		
Dexamethasone-containing	69 (81)	68 (82)
Prior IMiD	21 (25)	21 (25)
Thalidomide-containing	14 (16)	11 (13)
Lenalidomide-containing	7 (8)	12 (14)
Bortezomib-containing	40 (47)	39 (47)
Melphalan-containing	61 (72)	62 (75)
Cyclophosphamide-containing	29 (34)	29 (35)
Prednisolone-containing	3 (4)	4 (5)
Bendamustine-containing	1 (1)	0
Other	7 (8)	2 (2)

All values are *n* (%) unless otherwise indicated.

*ECOG* Eastern Cooperative Oncology Group, *IMiD* immunomodulatory drug, *NYHA* New York Heart Association, *PI* proteasome inhibitor, *SCT* stem cell transplant.

^a^Race not reported in 3 patients in the ixazomib–dexamethasone arm.

^b^Patients could report multiple sites of amyloid involvement.

^c^A subgroup of the Mayo 2004 [5] cardiac risk stage 3 was used in which both NT-proBNP and troponin T were over the respective thresholds of NT-proBNP >332 pg/mL and troponin T > 0.035 ng/mL, but NT-proBNP was <8000 pg/mL.

### Efficacy

**Fig. 1:**
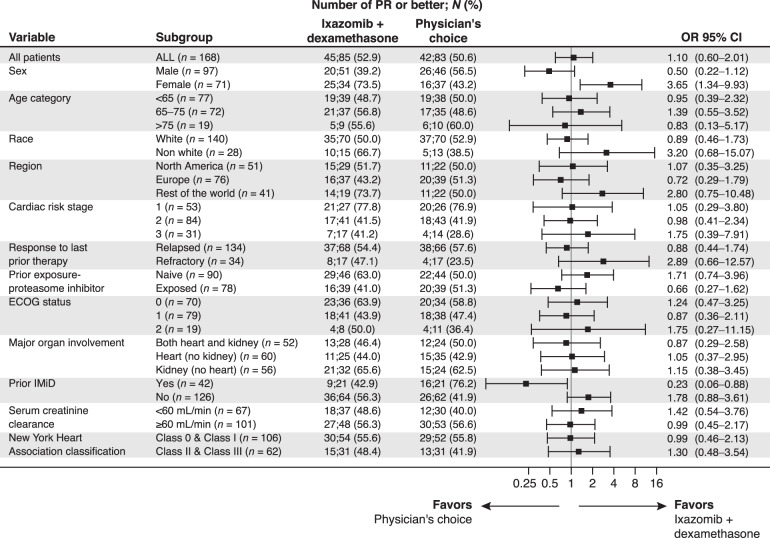
Forest plot of overall hematologic response rate by baseline characteristics (intent-to-treat population). *CI* confidence interval, *ECOG* Eastern Cooperative Oncology Group, *IMiD* immunomodulatory drug, *OR* odds ratio, *PR* partial response.

At data cut-off (February 20, 2019), all patients had completed 6 cycles of treatment or discontinued beforehand, and IA1 was performed. Best hematologic responses were seen in 53% (45/85; 95% CI, 41.8–63.9) of ixazomib–dexamethasone vs 51% (42/83; 95% CI, 39.4–61.8) of physician’s choice patients (unstratified CMH *p* = 0.76); the first primary endpoint was therefore not met. Hematologic CR rate (key secondary endpoint) was 26% (22/85) vs 18% (15/83) (odds ratio, 1.58; 95% CI, 0.76–3.32, *p* = 0.22; Table 2). Hematologic responses by physician’s choice of treatment are presented in Supplementary Table 1 and by baseline characteristics in Fig. 1. Response rate was 63% (29/46) vs 50% (22/44) in PI-naïve patients (favoring ixazomib–dexamethasone) and 41% (16/39) vs 51% (20/39) (favoring physician’s choice) in PI-exposed patients (Table 3). Among all patients, median duration of hematologic response was 46.5 months (ixazomib–dexamethasone) vs 20.2 months (physician’s choice) (HR, 0.55; 95% CI, 0.26–1.18; *p* = 0.12).

**Table 2:** Hematologic and organ response (intent-to-treat population).

Best response^a^	Ixazomib–dexamethasone (*n* = 85)	Physician’s choice (*n* = 83)	Odds ratio [95% CI^b^], *p* value^c^
Hematologic response			
PR or better (PR + VGPR + CR)	45 (53) [41.8–63.9]	42 (51) [39.4–61.8]	1.10 [0.60–2.01], *p* = 0.76
CR	22 (26) [17.0–36.5]	15 (18) [10.5–28.0]	1.58 [0.76–3.32], *p* = 0.22
VGPR	14 (16) [9.3–26.1]	17 (20) [12.4–30.8]	
PR	9 (11) [5.0–19.2]	10 (12) [5.9–21.0]	
No change^d^	35 (41) [30.6–52.4]	33 (40) [29.2–51.1]	
PD	2 (2) [0.3–8.2]	0	
Missing^e^	3 (4) [0.7–10.0]	8 (10) [4.3–18.1]	
Vital organ			
Response	31 (36) [26.3–47.6]	9 (11) [5.1–19.6]	4.72 [2.08–10.73] *p* = 0.0001
Stable disease	46 (54) [43.0–65.0]	58 (70) [58.8–19.5]	
Progression	3 (4) [0.7–10.0]	6 (7) [2.7–15.1]	
Missing^e^	5	10	
Cardiac response only			
Response	15 (18) [10.2–27.4]	4 (5) [1.3–11.9]	4.23 [1.34–13.35] *p* = 0.0089
Stable disease	48 (56) [45.3–67.2]	52 (63) [51.3–73.0]	
Progression	7 (8) [3.4–16.2]	7 (8) [3.5–16.6]	
Missing^e^	15	20	
Renal response only			
Response	24 (28) [19.0–39.0]	6 (7) [2.7–15.1]	5.05 [1.94–13.13] *p* = 0.0004
Stable disease	41 (48) [37.3–59.3]	49 (59) [47.7–69.7]	
Progression	3 (4) [0.7–10.0]	4 (5) [1.3–11.9]	
Missing^e^	17	24	

*CI* confidence interval, *CR* complete response, *PD* progressive disease, *PR* partial response, *VGPR* very good partial response.

^a^All values are shown as *n* (%) [95% CI], with 95% CI based on exact binomial distributions.

^b^Odds ratio calculated from a logistic regression model; 95% CI based on the Wald approximation. An odds ratio of >1 indicates ixazomib–dexamethasone is superior to physician’s choice.

^c^*p* value calculated from the unstratified Cochran–Mantel–Haenszel test.

^d^No CR, VGPR, PR, no progression.

^e^Missing values were defined as no post-baseline response assessment either due to loss to follow-up or patient withdrawal. If the response assessment was missing, it was counted as a failure (nonresponder) instead of a missing value.

**Table 3:** Hematologic response by prior PI exposure.

	Ixazomib–dexamethasone (*n* = 85)	Physician’s choice (*n* = 83)	Odds ratio [95% CI^b^], *p* value^c^
PI-naïve	*n* = 46	*n* = 44	
PR or better (PR + VGPR + CR)	29 (63) [47.5–76.8]	22 (50) [34.6–65.4]	1.71 [0.74–3.96], *p* = 0.21
CR	15 (33) [31.3–68.7]	12 (27) [29.1–70.9]	
VGPR	7 (15) [23.0–77.0]	7 (16) [23.0–77.0]	
PR	7 (15) [23.0–77.0]	3 (7) [11.8–88.2]	
No change^d^	14 (30) [30.6–69.4]	15 (34) [31.3–68.7]	
PD	0	0	
PI-exposed	*n* = 39	*n* = 39	
PR or better (PR + VGPR + CR)	16 (41) [25.6–57.9]	20 (51) [34.8–67.6]	0.66 [0.27–1.62] *p* = 0.37
CR	7 (18) [23.0–77.0]	3 (8) [11.8–88.2]	
VGPR	7 (18) [23.0–77.0]	10 (26) [27.2–72.8]	
PR	2 (5) [6.8–93.2]	7 (18) [23.0–77.0]	
No change^d^	21 (54) [34.2–65.8]	18 (46) [32.9–67.1]	
PD	2 (5) [6.8–93.2]	0	

*CI* confidence interval, *CR* complete response, *PD* progressive disease, *PI* proteasome inhibitor, *PR* partial response, *VGPR* very good partial response.

^a^All values are shown as *n* (%) [95% CI], with 95% CI based on exact binomial distributions.

^b^Odds ratio calculated from a logistic regression model; 95% CI based on the Wald approximation. An odds ratio of >1 indicates ixazomib–dexamethasone is superior to physician’s choice.

^c^*p* value calculated from the unstratified Cochran–Mantel–Haenszel test.

^d^No CR, VGPR, PR, no progression.

**Fig. 2:**
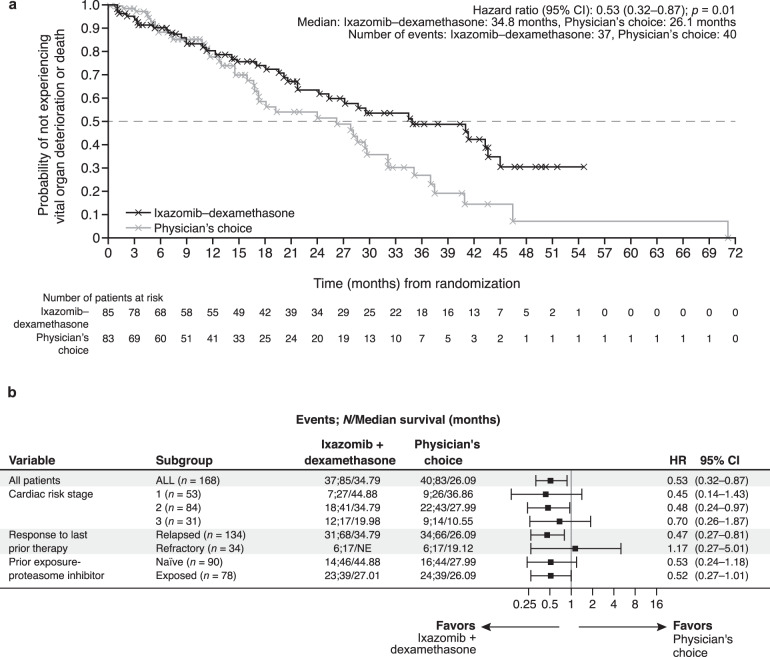
Time to vital organ deterioration or mortality in the intent-to-treat population. **a** Time to vital organ deterioration or mortality (investigator assessed)—defined as time from randomization to vital organ (heart or kidney) deterioration (evaluated according to central laboratory results and International Society of Amyloidosis criteria) or death, whichever occurred first. Cardiac deterioration was defined as the need for hospitalization for heart failure. Kidney deterioration was defined as progression to end-stage renal disease with the need for maintenance dialysis or renal transplantation. Patients without documentation of organ deterioration or death were censored at the date of the last assessment. **b** Time to vital organ deterioration and mortality by randomization stratification factors (investigator assessed). *CI* confidence interval, *HR* hazard ratio, *NE* not estimable.

Best vital organ responses occurred in 36% (31/85) of ixazomib–dexamethasone and 11% (9/83) of physician’s choice patients; cardiac-only response rate was 18% (15/85) vs 5% (4/83) and renal-only response rate was 28% (24/85) vs 7% (6/83) (Table 2). Two-year vital organ deterioration and mortality rate (second primary endpoint) data were immature; 47 (30%) patients had not yet completed 2-year follow-up. Time to vital organ deterioration or mortality was longer with ixazomib–dexamethasone than physician’s choice (median, 34.8 vs 26.1 months; HR, 0.53; 95% CI, 0.32–0.87; *p* = 0.01) (Fig. 2a and by stratification factors in Fig. 2b). Among the subset of patients with 2 years of follow-up, 40% (26/65) of ixazomib–dexamethasone and 45% (25/56) of physician’s choice patients had a vital organ deterioration or mortality event.

**Fig. 3:**
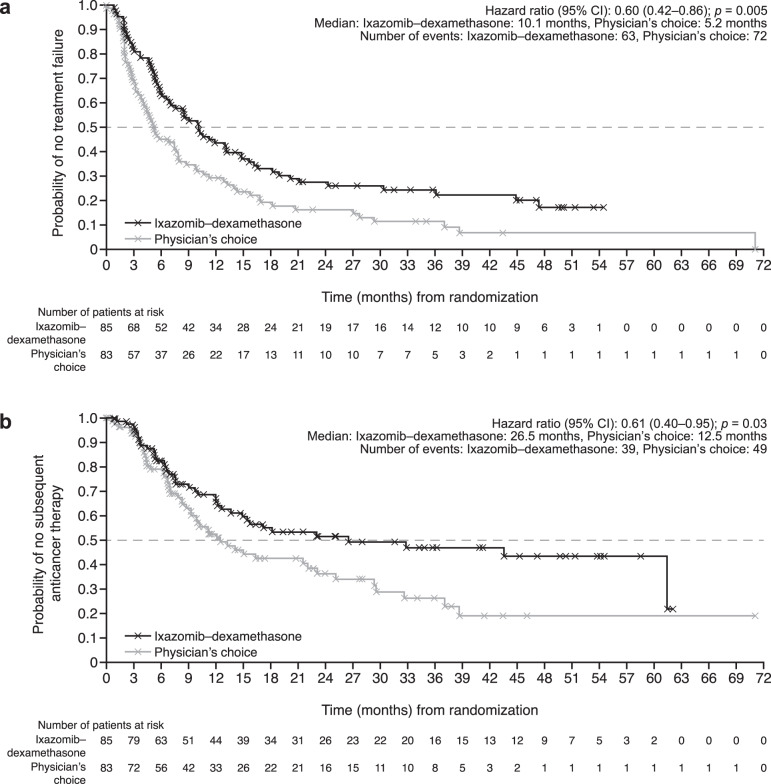
Time-to-event outcomes in the intent-to-treat population. **a** Time to treatment failure—defined as time from randomization to death due to any cause, hematologic or major organ progression (evaluated according to central laboratory results and International Society of Amyloidosis criteria), clinically morbid organ disease requiring additional therapy, or withdrawal for any reason. Patients without documentation of treatment failure were censored at the date of last response assessment. **b** Time to subsequent therapy—defined as time from randomization to the start of subsequent anticancer treatment. Patients without subsequent anticancer therapy were censored at the date of death or the last date they were known to be alive. *CI* confidence interval, *HR* hazard ratio.

**Fig. 4:**
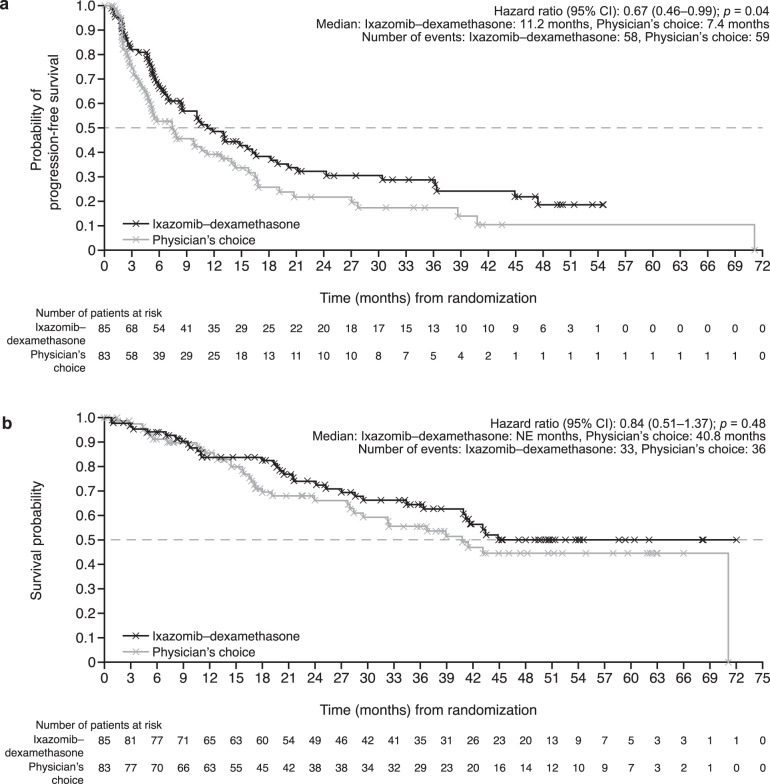
Survival endpoints in the intent-to-treat population. **a** Overall (hematologic and/or vital organ) progression-free survival (investigator assessed)—defined as time from randomization to first documentation of hematologic disease progression or vital organ (heart or kidney) progression, or death due to any cause, whichever occurred first. Patients without documentation of hematologic disease progression and organ progression were censored at the date of last hematologic response assessment that was stable disease or better, or the date of last organ assessment of stable disease or better, whichever occurred last. **b** Overall survival—defined as time from randomization to date of death. Patients without documentation of death at the time of analysis were censored at the date last known to be alive. *CI* confidence interval, *HR* hazard ratio, *NE* not estimable.

Median time to treatment failure was 10.1 months (ixazomib–dexamethasone) vs 5.2 months (physician’s choice) (HR, 0.60; 95% CI, 0.42–0.86; *p* = 0.005) (Fig. 3a); median time to subsequent therapy was 26.5 vs 12.5 months (HR, 0.61; 95% CI, 0.40–0.95; *p* = 0.03) (Fig. 3b). Median overall PFS was 11.2 vs 7.4 months (HR, 0.67; 95% CI, 0.46–0.99; *p* = 0.04) (Fig. 4a), hematologic PFS was 29.5 vs 27.7 months (HR, 0.76; 95% CI, 0.48–1.21; *p* = 0.24), and organ PFS was 18.0 vs 11.0 months (HR, 0.62; 95% CI, 0.41–0.93; *p* = 0.02). After 45.3 months median OS follow-up for ixazomib–dexamethasone and 43.4 months for physician’s choice, median OS was not reached vs 40.8 months (HR, 0.84; 95% CI, 0.51–1.37; *p* = 0.48) (Fig. 4b).

Time-to-event outcomes by prior PI exposure are shown in Supplementary Fig. 2. Median time to vital organ deterioration or mortality in PI-naïve patients was 44.9 months (ixazomib–dexamethasone *n* = 46) vs 28.0 months (physician’s choice *n* = 44) (HR, 0.53) and 27.0 vs 26.1 months in PI-exposed patients (*n* = 39 vs *n* = 39; HR, 0.52). Median OS in PI-naïve patients was not reached (ixazomib–dexamethasone) vs 71.1 months (physician’s choice) (HR, 0.81), and 40.9 vs 32.4 months in PI-exposed patients (HR, 0.85). HRs were 0.46–0.85 in favor of ixazomib–dexamethasone vs physician’s choice in both PI-naïve and PI-exposed patients.

### Safety

Median treatment duration was 11.7 months (ixazomib–dexamethasone) vs 5.0 months (physician’s choice) (HR, 0.46; 95% CI, 0.32–0.67). Of 24 patients receiving melphalan–dexamethasone, 4 stopped treatment according to protocol for melphalan. The safety population comprised all ixazomib–dexamethasone patients and 81 physician’s choice patients (Supplementary Fig. 1). Safety is summarized in Supplementary Table 2**;** grade ≥3 AEs were experienced by 62% (53/85) of ixazomib–dexamethasone and 56% (45/81) of physician’s choice patients. Any-grade AEs of clinical importance in ixazomib–dexamethasone versus physician’s choice patients included diarrhea (34 vs 30%), rash (33 vs 20%), cardiac arrhythmias (26 vs 15%, of which 8 vs 1% were due to non-drug-related atrial fibrillation), nausea (24 vs 14%), pneumonia (21 vs 16%), and peripheral neuropathy (19 vs 15%) (Table 4). The most common (>5% overall) grade ≥3 AEs were fatigue (9% in both arms), anemia (2 vs 11%), and cardiac failure and dyspnea (each 6 vs 4%). Events by total person years on treatment were 544 with ixazomib–dexamethasone vs 1400 with physician’s choice.

**Table 4:** Most common any-grade (reported in ≥10% of patients in either arm) and grade ≥3 AEs, and AEs of clinical importance (safety population).

Preferred term	Ixazomib–dexamethasone (*n* = 85)		Physician’s choice (*n* = 81)	
	Any grade	Grade ≥ 3	Any grade	Grade ≥ 3
Fatigue	38 (45)	8 (9)	35 (43)	7 (9)
Peripheral edema	39 (46)	4 (5)	26 (32)	4 (5)
Diarrhea	29 (34)	2 (2)	24 (30)	2 (2)
Insomnia	32 (38)	3 (4)	14 (17)	2 (2)
Constipation	18 (21)	1 (1)	21 (26)	3 (4)
Dyspnea	20 (24)	5 (6)	15 (19)	3 (4)
Upper respiratory tract infection	20 (24)	1 (1)	13 (16)	0
Nausea	20 (24)	1 (1)	11 (14)	0
Peripheral sensory neuropathy	16 (19)	1 (1)	10 (12)	1 (1)
Dizziness	13 (15)	0	11 (14)	0
Vomiting	13 (15)	1 (1)	11 (14)	0
Anemia	7 (8)	2 (2)	15 (19)	9 (11)
Decreased appetite	10 (12)	1 (1)	9 (11)	0
Back pain	15 (13)	1 (1)	8 (10)	1 (1)
Muscle spasms	9 (11)	0	9 (11)	0
Muscular weakness	13 (15)	0	5 (6)	1 (1)
Asthenia	9 (11)	3 (4)	8 (10)	2 (2)
Cough	10 (12)	0	6 (7)	0
Abdominal distension	10 (12)	0	4 (5)	0
Epistaxis	10 (12)	0	4 (5)	0
Headache	10 (12)	0	4 (5)	0
Rash maculo-papular	11 (13)	3 (4)	3 (4)	2 (2)
Arthralgia	10 (12)	0	3 (4)	1 (1)
Hypertension	10 (12)	1 (1)	2 (2)	1 (1)
Bronchitis	9 (11)	0	1 (1)	1 (1)
Pain in extremity	9 (11)	0	1 (1)	0
Other AEs of clinical importance				
Rash	28 (33)	3 (4)	16 (20)	4 (5)
Cardiac arrhythmias	22 (26)	8 (9)	12 (15)	5 (6)
Atrial fibrillation	7 (8)	1 (1)	1 (1)	0
Pneumonia	18 (21)	4 (5)	13 (16)	8 (10)
Peripheral neuropathy	16 (19)	1 (1)	12 (15)	1 (1)

*AE* adverse event.

All values are shown as *n* (%).

SAEs were reported in 47% (40/85) of ixazomib–dexamethasone vs 33% (27/81) of physician’s choice patients; SAEs occurring in ≥5% of patients in either arm were pneumonia (5 vs 5%) and dyspnea (5 vs 1%). AEs resulting in discontinuation were reported in 26% (22/85) vs 20% (16/81) of patients. Five patients (6%) receiving ixazomib–dexamethasone and 4 (5%) receiving physician’s choice died on-study (Supplementary Table 2); all deaths were considered related to AL amyloidosis or complications thereof, and all patients had pre-existing cardiac disease.

## Discussion

Randomized controlled trials in AL amyloidosis are scarce due to the rarity of the disease, and there are no standard primary endpoints for AL amyloidosis. TOURMALINE-AL1 is the first randomized phase 3 trial to be conducted in relapsed/refractory AL amyloidosis. It provides clinically meaningful data for patients and physicians that can inform treatment choices and further scientific understanding of this rare disease, and it also highlights the challenges associated with clinical trial design and recruitment in this setting. All time-to-event data favored ixazomib–dexamethasone over physician’s choice regimens. Ixazomib–dexamethasone was well tolerated (no new safety signals seen compared to prior studies in AL amyloidosis and relapsed/refractory MM) [24, 27], enabling twice as long a treatment duration vs physician’s choice.

As the first phase 3 trial in this setting, several elements of the study design are noteworthy. This trial was designed in 2012, working collaboratively with regulatory agencies and the amyloidosis medical community to design a clinically meaningful study for this patient population. The novel composite endpoint of 2-year vital organ deterioration and mortality rate was formulated despite a lack of historical data on which to base statistical assumptions. Assumptions relating to power had to be based on data from small, single-arm studies, many of which had enrolled both treatment-naïve and relapsed/refractory patients. Furthermore, hematologic response rate was chosen as the first primary endpoint based on feedback from experts in the field, as an endpoint believed to be a surrogate for clinical benefit. At the time of study design, however, hematologic response rate was being validated in newly diagnosed rather than relapsed/refractory AL amyloidosis. This study could have helped validate it in relapsed/refractory patients [28]. The choice of control arm was complicated by the absence of agreed standards of care because there were no preceding randomized trials in the relapsed setting. The choice of control arm was determined based upon the consensus of the best available options, globally, at the time of trial design, according to amyloidosis experts’ advice. Thus, an additive trial design per those conventionally used in multiple myeloma—of investigating novel doublet versus single-agent, or novel triplet versus standard-of-care doublet—was not feasible at the time in this setting. Over the course of the trial, which was prolonged due to the rarity of the disease and the difficulty in recruiting patients who were bortezomib-naïve hindering accrual, the widespread and increased application of approved multiple myeloma regimens may have in part affected outcomes. The benefit and tolerability ixazomib–dexamethasone demonstrated in PI-exposed patients suggest that this regimen may represent a valid option in relapsed-refractory patients previously exposed to daratumumab and bortezomib.

In the treatment of newly diagnosed AL amyloidosis, deeper hematologic responses are associated with improved organ response and OS, and organ responses are linked with improved prognosis [3, 11, 18, 19, 29, 30]. Responses were deeper (CR, 26 vs 18%) and more durable (median, 46.5 vs 20.2 months) with ixazomib–dexamethasone vs physicians’ choice, although the overall hematologic response rate was similar between arms (53 vs 51%). Consistently, best vital organ response rates were higher with ixazomib–dexamethasone vs physician’s choice (36 vs 11%), although organ response assessment based on NT-proBNP levels per ISA criteria may have underestimated rates in patients receiving lenalidomide–dexamethasone due to NT-proBNP-increases observed with immunomodulatory drugs [31].

Notably, the hematologic response reported with ixazomib–dexamethasone is consistent with the 52% rate reported in the phase 1/2 study of ixazomib in relapsed/refractory AL amyloidosis in which patients with less than a partial response after 3 cycles received added dexamethasone [24]. Historically, hematologic response rates with dexamethasone alone are 35–40% (albeit using different response criteria and including both newly diagnosed and pretreated patients [32, 33]), suggesting a clear treatment effect with ixazomib. For comparison, hematologic response rates of 39–69% have been reported for single-agent bortezomib in PI-naïve relapsed AL amyloidosis [34]. In this phase 3 study, hematologic responses by regimen in the physician’s choice arm were consistent (range, 50–58%), except for cyclophosphamide–dexamethasone (30%), although the small numbers of patients preclude meaningful conclusions. Similarly, small subgroups and wide confidence intervals did not allow confirmation of any differences in hematologic response rates according to patients’ baseline characteristics. The statistical assumption of a 40% hematologic response rate for physician’s choice was based on data from single-arm studies [35–41] (which often included a mix of newly diagnosed and relapsed patients) and assumed equal enrollment across all regimens. The fact that no patients received a physician’s choice of dexamethasone alone and that nearly a third of patients received the melphalan and dexamethasone combination, which resulted in a 58% response rate (predominant in countries where melphalan would not have been available outside of this clinical trial), and over half of patients received lenalidomide plus dexamethasone, which resulted in a 51% response rate (predominant in countries where lenalidomide would not have been available outside of this clinical trial) could account for the higher-than-expected response rate. Since an additive trial design was not feasible at the time of study design, this resulted in the ixazomib–dexamethasone doublet being compared against two effective doublets, melphalan plus dexamethasone and lenalidomide plus dexamethasone, as a result of the evolving standards of care in the relapsed/refractory AL amyloidosis setting.

The second primary endpoint of the rate of 2-year vital organ deterioration and mortality was lower with ixazomib–dexamethasone vs physician’s choice (40 vs 45%) in evaluable patients. Further, time to vital organ deterioration and mortality was numerically longer with ixazomib–dexamethasone vs physician’s choice (events 37 vs 40; HR, 0.53; 95% CI, 0.32–0.87). With only 40% of OS events reached, there was a trend for an improvement in OS (HR, 0.84; 95% CI, 0.51–1.37). While the hematologic response rate in PI-exposed patients favored treatment with physician’s choice, the HRs for longer-term outcomes of time to vital organ deterioration and mortality and OS favored ixazomib–dexamethasone in both PI-naïve and PI-exposed populations. The magnitude of benefit with ixazomib–dexamethasone vs physician’s choice was greater in PI-naïve patients vs PI-exposed patients, which is expected based on the previous phase 1/2 study of ixazomib (± dexamethasone) in relapsed/refractory AL amyloidosis in which PI-naïve patients had a median hematologic PFS of 25.8 months compared with 10.7 months in PI-exposed patients [24]. It is also consistent with a phase 2 study of ixazomib–dexamethasone in relapsed MM in which the median event-free survival in bortezomib-naïve patients was double that in bortezomib-exposed patients [42]. The benefit demonstrated with ixazomib–dexamethasone vs physician’s choice in PI-exposed patients in this phase 3 study, provides important information for patients who have previously received a PI and their treating physicians. This represents a clinically relevant benefit in this setting, given the paucity of treatment options with no standard of care available. Across all patients, all other time-to-event endpoints including overall and organ PFS, time-to-treatment failure, and time to subsequent therapy were numerically longer with ixazomib–dexamethasone over physician’s choice.

Safety was similar between the 2 arms, with AEs reported for ixazomib–dexamethasone consistent with the known ixazomib safety profile [24, 42, 43]. Rash and peripheral neuropathy were more common with ixazomib–dexamethasone, but events were mainly low-grade. The increase in cardiac arrhythmias in the ixazomib–dexamethasone arm (26 vs 15%) is thought to be a result of the higher frequency of non-drug-related atrial fibrillation in patients with cardiac involvement (8 vs 1%) or longer exposure to drug. Numerically higher rates of SAEs and AEs resulting in discontinuation were reported with ixazomib–dexamethasone vs physician’s choice; however, this was not surprising given that patients received ixazomib–dexamethasone for twice as long as physician’s choice. On-study deaths were similar between arms; none were considered related to treatment.

Long-term treatment with certain PIs may be challenging for some patients due to burden of administration, comorbidities, organ dysfunction, or toxicity [12, 15]. Peripheral neuropathy is a known side effect with bortezomib and, as such, bortezomib is not recommended for patients with existing grade 3–4 neuropathy [15]; although rates of such events appear reduced with subcutaneous dosing [44]. Carfilzomib has been investigated in a phase 1/2 study in relapsed/refractory AL amyloidosis. Hematologic response rate was 63%; however, cardiac, pulmonary, and renal toxicities were common, and may limit the use of carfilzomib in this setting [45]. Data from TOURMALINE-AL1 demonstrate that ixazomib–dexamethasone is generally well tolerated in AL amyloidosis with a clinically relevant treatment duration (enabling deeper hematologic responses and higher organ response rates than physician’s choice), thereby providing patients with a potential longer-term PI-based treatment option.

TOURMALINE-AL1, the first phase 3 trial in relapsed/refractory AL amyloidosis, demonstrates outcome improvements with ixazomib–dexamethasone vs physician’s choice in evaluable patients. Ixazomib–dexamethasone prolonged time to vital organ deterioration and mortality, PFS, and time to subsequent therapy vs physician’s choice in patients with relapsed/refractory AL amyloidosis. Ixazomib–dexamethasone also improved the depth and duration of response, although the first primary endpoint of overall hematologic response rate was not met. Treatment with ixazomib–dexamethasone was generally well tolerated, enabling patients to receive treatment for twice as long as physician’s choice. The time-to-event data suggest that ixazomib–dexamethasone may benefit patients with relapsed/refractory AL amyloidosis who have limited treatment options.

## Supplementary information

Supplementary Appendix

## Data Availability

The datasets, including the redacted study protocol, redacted statistical analysis plan, and individual participants’ data supporting the results reported in this article, will be made available within three months from initial request, to researchers who provide a methodologically sound proposal. The data will be provided after its de-identification, in compliance with applicable privacy laws, data protection and requirements for consent and anonymization.
