# Supplementary material for: A randomized phase 3 study of ixazomib–dexamethasone versus physician’s choice in relapsed or refractory AL amyloidosis
Source: Leukemia. 2021 Jun 24;36(1):225–35. doi: 10.1038/s41375-021-01317-y (PMC8727292; doi:10.1038/s41375-021-01317-y)
Supplement: Supplementary file 1 — Supplementary Appendix [file 41375_2021_1317_MOESM1_ESM.docx]

SUPPLEMENTARY APPENDIX

One Word document describing supplementary methods

2 supplementary figures submitted as PDFs

2 supplementary tables

## Supplementary methods

### Patient eligibility criteria

#### Inclusion criteria

- Male or female patients aged 18 years or older
- Biopsy-proven diagnosis of AL amyloidosis, according to the following standard criteria:
  - Histochemical diagnosis of amyloidosis, as based on tissue specimens with Congo red staining with exhibition of an apple-green birefringence
  - If clinical and laboratory parameters are insufficient to establish AL amyloidosis, or in cases of doubt, amyloid typing may be necessary
- Measurable disease, as defined by serum differential free light-chain concentration (dFLC; defined as the difference between amyloid forming [involved] and nonamyloid forming [uninvolved] free light-chain [FLC]) ≥50 mg/L)
- Objective, measurable major organ (cardiac or renal) amyloid involvement, defined as follows (amyloid involvement of ≥1 organ [cardiac or renal] is required):
  - Cardiac involvement is defined as the presence of a mean left ventricular wall thickness on echocardiogram of >12 mm in the absence of other potential causes of left ventricular hypertrophy (controlled hypertension is allowed) with a noncardiac biopsy showing amyloid, or a positive cardiac biopsy in the presence of clinical or laboratory evidence of involvement. If there is isolated cardiac involvement, then typing of amyloid deposits is recommended
  - Renal involvement is defined as proteinuria (predominantly albumin) >0.5 g/day in a 24-hour urine collection
  - Note: amyloid involvement of other organ systems is allowed, but not required
- Must be relapsed or refractory after 1 or 2 prior therapies. Relapsed is defined as documented progressive disease >60 days after the last dose of prior therapy and refractory is defined as the documented absence of a hematologic response or hematologic progression on or within 60 days after the last dose of prior therapy
  - The patient could have been previously treated with proteasome inhibitors, but may not be refractory to proteasome inhibitor therapy
  - Given that the physician may select from an offered list of regimens to treat a specific patient, the patient may be refractory to an agent(s) listed within the list of offered treatment choices
  - The patients must have recovered (ie, grade ≤1 toxicity or patient’s baseline status) from the reversible effects of prior therapy
  - If a patient has received a transplant as their first-line therapy, they must be ≥3 months post transplantation and have recovered from the side effects of the stem cell transplant
- Patient must meet the criteria for 1 of the following Mayo 2004 AL amyloidosis risk stages (as defined by an NT-proBNP cut-off of 332 pg/mL and a troponin T cut-off of 0.035 ng/mL, as thresholds) [1]:
  - Stage 1: both NT-proBNP and troponin T under the thresholds
  - Stage 2: either NT-proBNP or troponin T (but not both) over the thresholds
  - Stage 3: both NT-proBNP and troponin T over the thresholds (but NT-proBNP <8000 pg/mL)
- Eastern Cooperative Oncology Group performance status ≤2
- Clinical laboratory values:
  - Absolute neutrophil count ≥1000/µL
  - Platelet count ≥75,000/µL
  - Total bilirubin ≤1.5× the upper limit of normal (ULN), except for patients with Gilbert’s syndrome (as defined by >80% unconjugated bilirubin and total bilirubin ≤6 mg/dL)
  - Alkaline phosphatase ≤5× ULN
  - Alanine aminotransferase or aspartate aminotransferase ≤3× ULN
  - Calculated creatinine clearance ≥30 mL/min
- Female patients who:
  - If they are of childbearing potential, agree to practice 2 effective methods of contraception, at the same time, from the time of signing the informed consent through 90 days after the last dose of study treatment, AND
  - Must also adhere to the guidelines of any treatment-specific pregnancy prevention program, if applicable, OR
  - Agree to practice true abstinence when this is in line with the preferred and usual lifestyle of the patient (periodic abstinence [eg, calendar, ovulation, symptothermal, post-ovulation methods] and withdrawal are not acceptable methods of contraception)
- Male patients, even if surgically sterilized (ie, status post vasectomy), who:
  - Agree to practice effective barrier contraception during the entire study treatment period and through 90 days after the last dose of study drug, AND
  - Must also adhere to the guidelines of any treatment-specific pregnancy prevention program, if applicable, OR
  - Agree to practice true abstinence when this is in line with the preferred and usual lifestyle of the patient (periodic abstinence [eg, calendar, ovulation, symptothermal, post-ovulation methods] and withdrawal are not acceptable methods of contraception)
- Voluntary written consent must be given before performance of any study-related procedure not part of standard medical care with the understanding that consent may be withdrawn by the patient at any time without prejudice to future medical care

#### Exclusion criteria:

- Amyloidosis due to mutations of the transthyretin gene or presence of other non-AL amyloidosis
- Female patients who are lactating, breastfeeding, or pregnant
- Medically documented cardiac syncope, uncompensated New York Heart Association Class 3 or 4 congestive heart failure, myocardial infarction within the previous 6 months, unstable angina pectoris, clinically significant repetitive ventricular arrhythmias despite antiarrhythmic treatment, or severe orthostatic hypotension or clinically important autonomic disease
- Clinically overt multiple myeloma, according to the International Myeloma Working Group criteria, with ≥1 of the following:
  - Bone lesions
  - Hypercalcemia, defined as calcium >11 mg/dL
- Inability to swallow oral medication, inability or unwillingness to comply with the drug administration requirements, or a gastrointestinal procedure that could interfere with the oral absorption or tolerance of treatment
- Requirement for other concomitant chemotherapy, immunotherapy, radiotherapy, or any ancillary therapy considered to be investigational or which would be considered as treatment for AL amyloidosis. However, patients may be on chronic steroids (maximum dose, 20 mg/day prednisone or equivalent) if they are being given for disorders other than amyloidosis (eg, adrenal insufficiency, rheumatoid arthritis, etc.)
- Comorbid systemic illnesses or other severe concurrent disease which, in the judgment of the investigator, would make the patient inappropriate for entry into this study or interfere significantly with the proper assessment of the safety and toxicity of the prescribed regimens
- Ongoing or active infection, known HIV-positive status, or active hepatitis B or C infection
- Psychiatric illness or social situations that would limit compliance with study requirements
- Known allergy to boron, ixazomib, or any of the study treatments, their analogs or excipients
- Systemic treatment with strong CYP3A inducers (rifampin, rifapentine, rifabutin, carbamazepine, phenytoin, phenobarbital), or use of Ginkgo biloba or St. John’s wort within 14 days before the first dose of study treatment
- Diagnosed or treated for another malignancy within 3 years before study enrollment or previously diagnosed with another malignancy and have any evidence of residual disease. Patients with non-melanoma skin cancer or carcinoma in situ of any type are not excluded if they have undergone complete resection

**Definitions of primary and secondary endpoints.**

| **Endpoint** | **Definition** |
| --- | --- |
| **Primary** |  |
| Hematologic response rate | Overall hematologic (CR + VGPR + PR) response rate based on central laboratory results and the 2010 International Society of Amyloidosis (ISA) Consensus Criteria as evaluated by an Adjudication Committee (AC) |
| 2-year vital organ deterioration and mortality rate | 2-year vital organ (heart or kidney) deterioration and mortality rate. Cardiac deterioration is defined as the need for hospitalization for heart failure. Kidney deterioration is defined as progression to end-stage renal disease (ESRD) with the need for maintenance dialysis or renal transplantation. Vital organ deterioration will be evaluated by an AC. |
| **Key secondary** |  |
| Complete hematologic response rate (CR) | Complete hematologic response rate (CR) according to central laboratory results and ISA criteria as evaluated by an AC |
| Overall survival | Time from randomization to date of death. Patients without documentation of death at the time of analysis were censored at the date last known to be alive |
| Overall progression-free survival | Time from randomization to first documentation of hematologic disease progression* or vital organ (heart or kidney) progression*, or death due to any cause, whichever occurred first. Patients without documentation of hematologic disease progression and organ progression were censored at the date of last hematologic response assessment that was stable disease or better, or the date of last organ assessment of stable disease or better, whichever occurred last |
| Hematologic progression-free survival | Time from randomization to first documentation of hematologic disease progression* or death due to any cause, whichever occurred first. Patients without documentation of hematologic disease progression were censored at the date of last hematologic response assessment that was stable disease or better |
| Time to vital organ deterioration and mortality | Time from randomization to vital organ (heart or kidney) deterioration* or death, whichever occurred first. Cardiac deterioration was defined as the need for hospitalization for heart failure. Kidney deterioration was defined as progression to end-stage renal disease with the need for maintenance dialysis or renal transplantation. Patients without documentation of organ deterioration or death were censored at the date of the last assessment |
| Vital organ best response | Best response in the vital organs allowed at study entry (heart and kidney) according to central laboratory results and International Society of Amyloidosis criteria as  evaluated by an AC |
| Vital organ progression-free survival | Time from randomization to first documentation of vital organ (heart or kidney) progression* or death due to any cause, whichever occurred first. Patients without documentation of vital organ (heart or kidney) progression* were censored at the date of last organ assessment of stable disease or better |
| Duration of hematologic response | Time from the date of first documentation of hematologic response to the date of first documented hematologic disease progression, respectively according to central laboratory results and ISA criteria as determined by an AC |
| Safety | Adverse events (AEs), serious adverse events (SAEs), and assessments of clinical laboratory values |
| Time to treatment failure | Time from randomization to death due to any cause, hematologic or major organ progression*, clinically morbid organ disease requiring additional therapy, or withdrawal for any reason. Patients without documentation of treatment failure were censored at the date of last response assessment |
| Time to subsequent anticancer treatment | Time from randomization to the start of subsequent anticancer treatment. Patients without subsequent anticancer therapy were censored at the date of death or the last date they were known to be alive |

*Evaluated according to central laboratory results and International Society of Amyloidosis criteria.

*Assumptions for response rate for treatment arms*

The assumption of a 65% response rate for the ixazomib–dexamethasone arm was based on the response rate of 52% reported in the phase 1/2 study of ixazomib in relapsed/refractory AL amyloidosis [2]. In the phase 1/2 study, patients initially received single-agent ixazomib, with dexamethasone added after 3 cycles for those patients with less than a partial response. In the present study, patients received ixazomib in combination with dexamethasone from cycle 1, which was anticipated would enhance the response rate. The statistical assumption of a 40% overall hematologic response rate for the physician’s choice arm was based on data from single-arm studies (which often included a mix of newly diagnosed and relapsed patients) and assumed equal enrollment across all regimens [3–11].

**References**

1. Dispenzieri A, Gertz MA, Kyle RA, et al. Serum cardiac troponins and N-terminal pro-brain natriuretic peptide: a staging system for primary systemic amyloidosis. J Clin Oncol. 2004;22:3751–57.

2. Sanchorawala V, Palladini G, Kukreti V, et al. A phase 1/2 study of the oral proteasome inhibitor ixazomib in relapsed or refractory AL amyloidosis. Blood. 2017;130:597–605.

3. Gertz MA, Lacy MQ, Lust JA, et al. Phase II trial of high-dose dexamethasone for untreated patients with primary systemic amyloidosis. Med Oncol. 1999;16:104–109.

4. Palladini G, Anesi E, Perfetti V, et al. A modified high-dose dexamethasone regimen for primary systemic (AL) amyloidosis. Br J Haematol. 2001;113:1044–46.

5. Dispenzieri A, Lacy MQ, Geyer SM, et al. Low Dose Single Agent Thalidomide Is Tolerated in Patients with Primary Systemic Amyloidosis, but Responses Are Limited. Blood. 2004;104:4920.

6. Dispenzieri A, Lacy MQ, Rajkumar SV, et al. Poor tolerance to high doses of thalidomide in patients with primary systemic amyloidosis. Amyloid. 2003;10:257–61.

7. Dispenzieri A, Lacy MQ, Zeldenrust SR, et al. The activity of lenalidomide with or without dexamethasone in patients with primary systemic amyloidosis. Blood. 2007;109:465–70.

8. Palladini G, Milani P, Foli A, et al. Melphalan and dexamethasone with or without bortezomib in newly diagnosed AL amyloidosis: a matched case-control study on 174 patients. Leukemia. 2014;28:2311–16.

9. Palladini G, Perfetti V, Perlini S, et al. The combination of thalidomide and intermediate-dose dexamethasone is an effective but toxic treatment for patients with primary amyloidosis (AL). Blood. 2005;105:2949–51.

10. Palladini G, Russo P, Foli A, et al. Salvage therapy with lenalidomide and dexamethasone in patients with advanced AL amyloidosis refractory to melphalan, bortezomib, and thalidomide. Ann Hematol. 2012;91:89–92.

11. Sanchorawala V, Wright DG, Rosenzweig M, et al. Lenalidomide and dexamethasone in the treatment of AL amyloidosis: results of a phase 2 trial. Blood. 2007;109:492–96.

**Supplementary Table 1.** Hematologic response in the physician’s choice arm by treatment regimen (intent-to-treat population)

| Best response^a^ | Dexamethasone–melphalan (*n* = 24) | Dexamethasone–cyclophosphamide (*n* = 10) | Dexamethasone–thalidomide (*n* = 2) | Dexamethasone–lenalidomide (*n* = 47) |
| --- | --- | --- | --- | --- |
| PR or better (PR+VGPR+CR) | 14 (58) [36.6–77.9] | 3 (30) [6.7–65.2] | 1 (50) [1.3–98.7] | 24 (51) [36.1–65.9] |
| CR | 6 (25) [9.8–46.7] | 0 | 1 (50) [1.3–98.7] | 8 (17) [7.6–30.8] |
| VGPR | 5 (21) [7.1–42.2] | 3 (30) [6.7–65.2] | 0 | 9 (19) [9.1–33.3] |
| PR | 3 (13) [2.7–32.4] | 0 | 0 | 7 (15) [6.2–28.3] |
| No change^b^ | 8 (33) [15.6–55.3] | 7 (70) [34.8–93.3] | 0 | 18 (38) [24.5–53.6] |
| PD | 0 | 0 | 0 | 0 |
| Missing^c^ | 2 (8) [1.0–27.0] | 0 | 1 (50) [1.3–98.7] | 5 (11) [3.5–23.1] |

*CI* confidence interval, *CR* complete response, *PD* progressive disease, *PR* partial response, *VGPR* very good partial response.

^a^Values are shown as *n* (%) [95% CI], with 95% CI based on exact binomial distributions.
^b^No CR, VGPR, PR, no progression. Missing values were defined as no post-baseline hematologic response assessment either due to loss to follow-up or patient withdrawal. If the hematologic response assessment was missing, it was counted as a failure (nonresponder) instead of a missing value.

**Supplementary** **Table 2.** Summary of safety (safety population)

| Adverse events, *n* (%) | Ixazomib–dexamethasone  (*n* = 85) | Physician’s choice (*n* = 81) |
| --- | --- | --- |
| Any adverse event | 84 (99) | 78 (96) |
| Any drug-related adverse event | 70 (82) | 66 (81) |
| Any grade ≥3 adverse event | 53 (62) | 45 (56) |
| Any drug-related grade ≥3 adverse event | 29 (34) | 33 (41) |
| Serious adverse events | 40 (47) | 27 (33) |
| Drug-related serious adverse event | 13 (15) | 13 (16) |
| Adverse events resulting in study drug discontinuation | 22 (26) | 16 (20) |
| On-study deaths | 5 (6) | 4 (5) |

**Supplementary** **Fig. 1** CONSORT flow diagram.


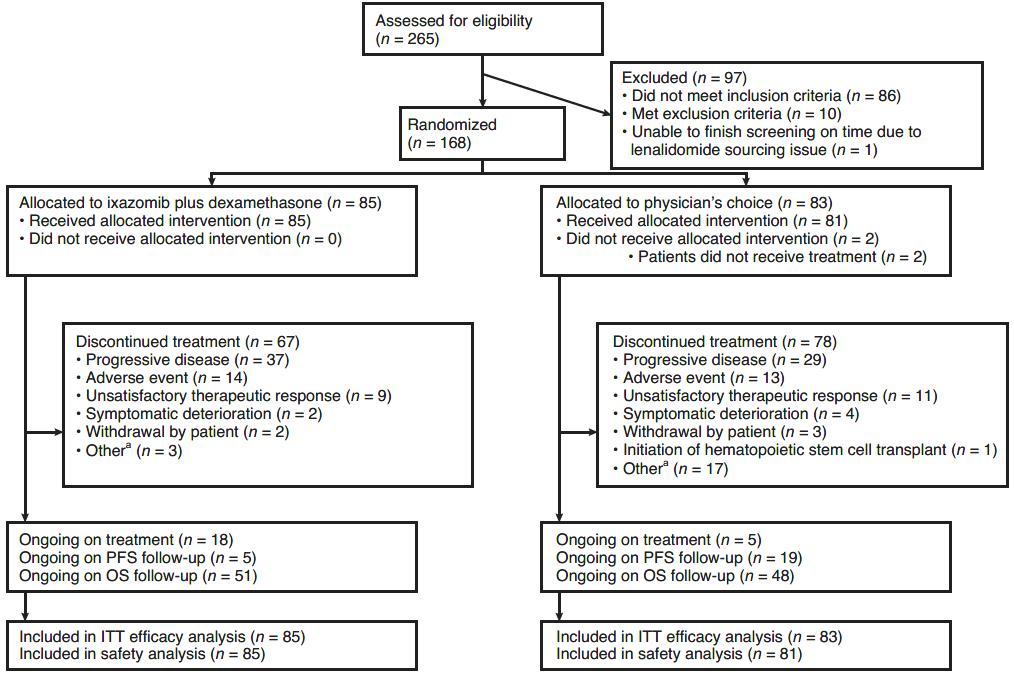


^a^“Other” includes physician/patient decision, reached maximal response or melphalan dosing, or additional medical procedure needed.

*ITT* intent to treat, *OS* overall survival, *PFS* progression-free survival.

**Supplementary Fig. 2** Kaplan–Meier analyses^a^ of **a** time to vital organ deterioration or mortality, **b** time to treatment failure, **c** time to subsequent therapy, **d** composite PFS, and **e** OS in PI-naïve (left) and PI-exposed (right) patients.


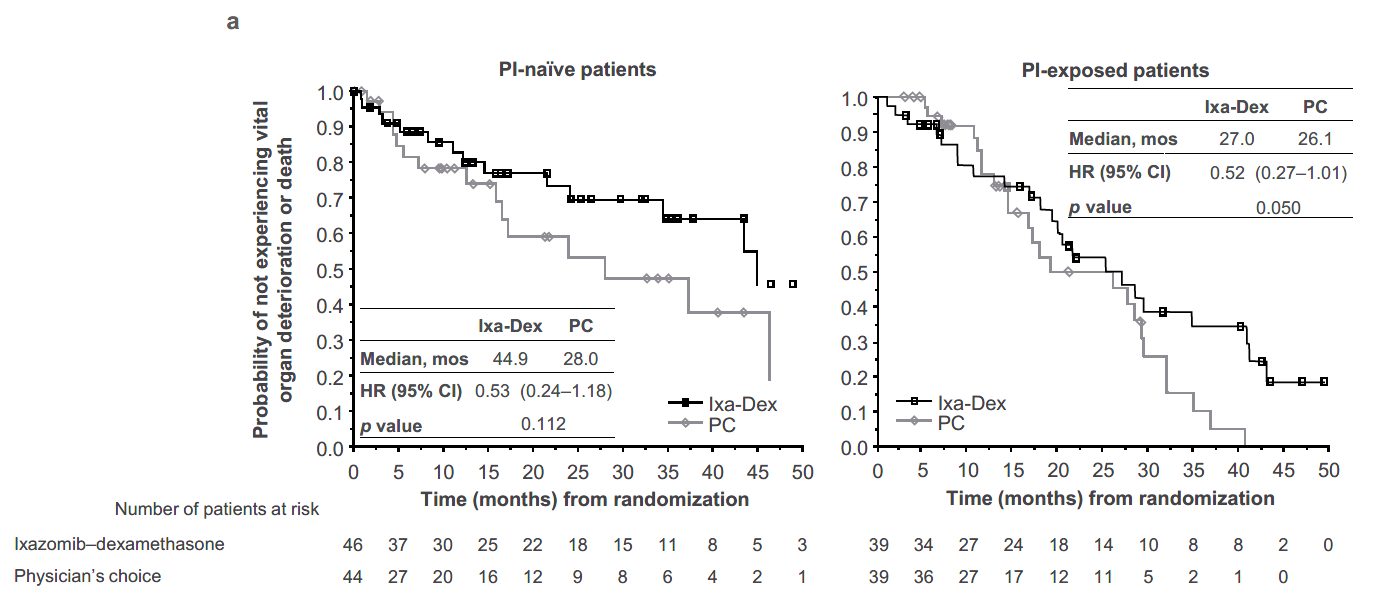


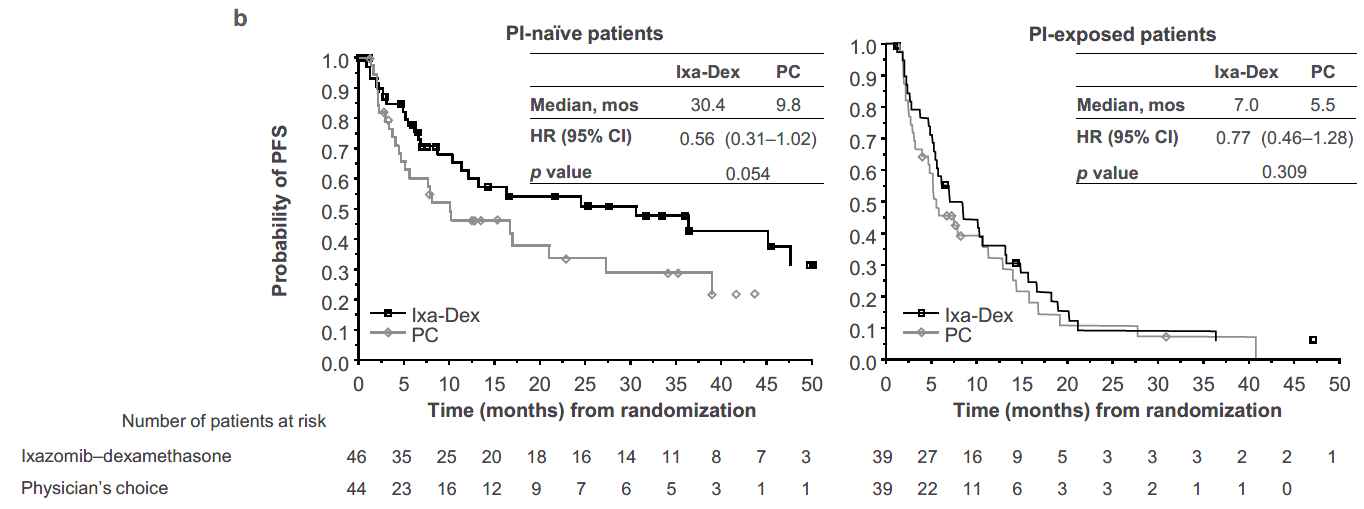


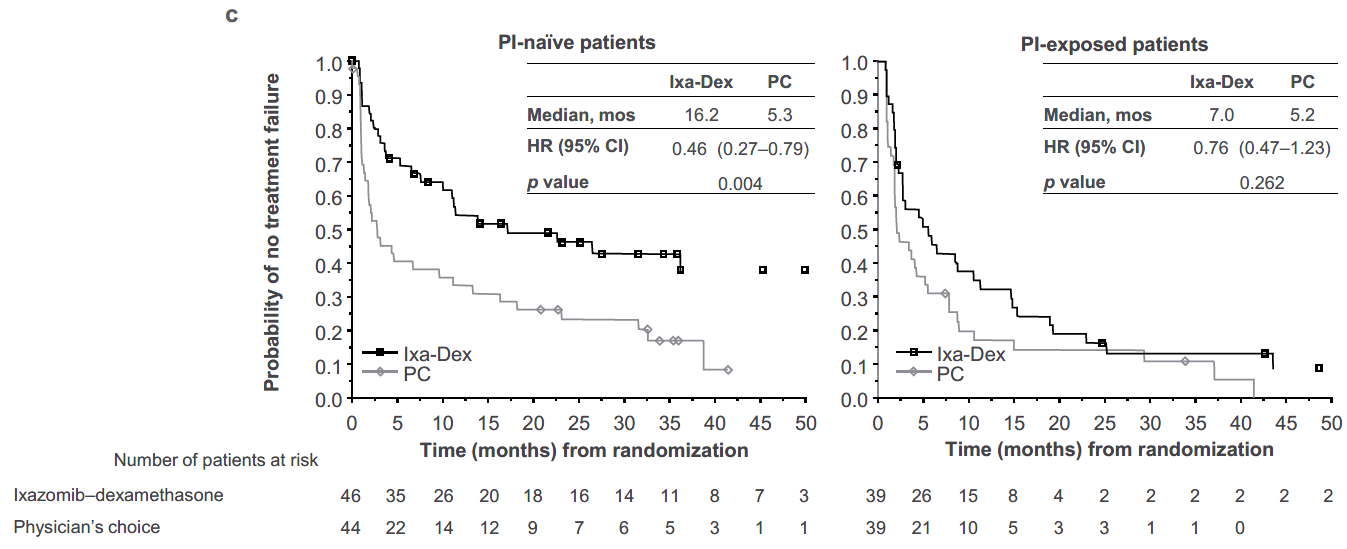


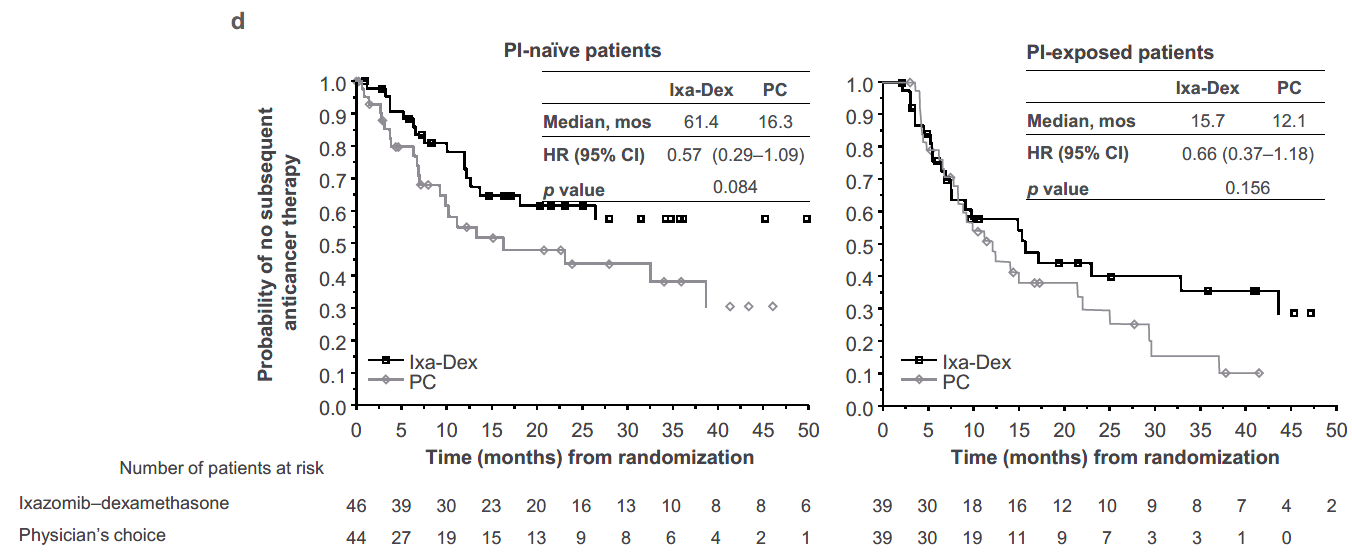


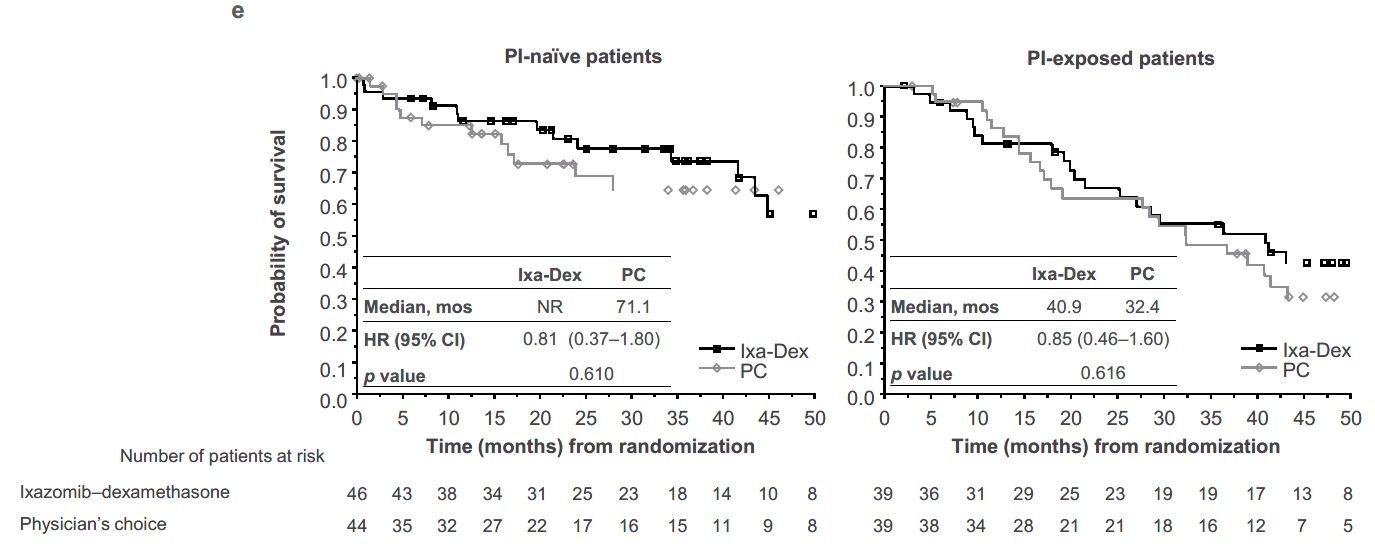


^a^A single HR to characterize the relative instantaneous risk of events is valid only when the proportional hazard (PH) assumption is valid. When the Kaplan-Meier curves cross, the PH assumption is unlikely to be valid. In those cases, the HR should be interpreted with caution.

*CI* confidence interval, *HR* hazard ratio, *ixa-dex* ixazomib–dexamethasone, *mos* months, *NR* not reached, *OS* overall survival, *PC* physician’s choice, *PFS* progression-free survival, *PH* proportional hazard, *PI* proteasome inhibitor.
